# A ceRNA-associated risk model predicts the poor prognosis for head and neck squamous cell carcinoma patients

**DOI:** 10.1038/s41598-021-86048-x

**Published:** 2021-03-18

**Authors:** Yuzi Xu, Fengqin Xu, Yiming Lv, Siyuan Wang, Jia Li, Chuan Zhou, Jimin Jiang, Binbin Xie, Fuming He

**Affiliations:** 1 Department of Oral Implantology and Prosthodontics, The Affiliated Hospital of Stomatology, School of Stomatology, Zhejiang University School of Medicine, and Key Laboratory of Oral Biomedical Research of Zhejiang Province, 395# Yanan Road, Hangzhou, 310006 Zhejiang People’s Republic of China; 2 Department of Medical Oncology, Sir Run Run Shaw Hospital, Zhejiang University School of Medicine, 3# East Qingchun Road, Hangzhou, 310016 Zhejiang People’s Republic of China; 3 The First Affiliated Hospital of Kangda College of Nanjing Medical University, The First People’s Hospital of Lianyungang, The Affiliated Lianyungang Hospital of Xuzhou Medical University, Lianyungang, 222000 Jiangsu People’s Republic of China; 4 Department of Colorectal Surgery, Sir Run Run Shaw Hospital, Zhejiang University School of Medicine, Hangzhou, 310016 Zhejiang People’s Republic of China

**Keywords:** Cancer, Computational biology and bioinformatics, Oncology

## Abstract

Head and neck squamous cell carcinoma (HNSCC) is one of the most malignant cancers with poor prognosis worldwide. Emerging evidence indicates that competing endogenous RNAs (ceRNAs) are involved in various diseases, however, the regulatory mechanisms of ceRNAs underlying HNSCC remain unclear. In this study, we retrieved differentially expressed long non-coding RNAs (DElncRNAs), messenger RNAs (DEmRNAs) and microRANs (DEmiRNAs) from The Cancer Genome Atlas database and constructed a ceRNA-based risk model in HNSCC by integrated bioinformatics approaches. Functional enrichment analyses showed that DEmRNAs might be involved in extracellular matrix related biological processes, and protein–protein interaction network further selected out prognostic genes, including MYL1 and ACTN2. Importantly, co-expressed RNAs identified by weighted co-expression gene network analysis constructed the ceRNA networks. Moreover, AC114730.3, AC136375.3, LAT and RYR3 were highly correlated to overall survival of HNSCC by Kaplan–Meier method and univariate Cox regression analysis, which were subsequently implemented multivariate Cox regression analysis to build the risk model. Our study provides a deeper understanding of ceRNAs on the regulatory mechanisms, which will facilitate the expansion of the roles on the ceRNAs in the tumorigenesis, development and treatment of HNSCC.

## Introduction

Head and neck squamous cell carcinoma (HNSCC) is one of the most common and malignant cancers with a yearly occurrence of 750,000 cases worldwide and 40–50% mortality^1,2^. HNSCC is characterized by a high propensity of recurrence and frequent metastasis developing in more than 65% of patients^3^ and second primary cancers at an annual rate of approximately 2–3%^4^. HNSCC can be staged according to tumor-node-metastasis (TNM) staging method, and the mainstays of therapeutic strategy is surgery in combination with radio- and chemotherapy for early-stage tumors^5^. Nevertheless, the survival rate of patients sharply declines once the disease has metastasized^6^. Systemic therapy with active agents including platinums, taxanes, antifolates, and cetuximab has remarkably improved the prognosis for a limited number of patients, thanks to the highlights of signature molecular markers^7^. For instance, Pavon et al. reported that overexpressed uPA/uPAR and SERPINE1 in HNSCC patients enhanced tumor cell proliferation, migration, and invasion^8^. And Hersi et al. discovered that plerixafor reversed the downregulation of miR‐9, hence suppressing cellular proliferation, cell cycle progression and colony formation^9^. However, the clinical efficacy of these biomarkers is hardly convincing, with low 5-year overall survival rates and high mortality rate^3^. As a result, it is of great importance to further investigate more effective biomarkers, signaling the incidence of HNSCC metastasis and poor prognosis, thus improving long-term curative effect.

The application of sequencing technology and the large-scale database have recently brought about novel approaches of cancer diagnosis, treatment and prognosis. Investigations about non-coding RNAs (ncRNAs) challenge the conventional perspectives on the predominant role of transcribed RNAs during protein synthesis^10^. Due to absence of open reading frames, ncRNAs such as long non-coding RNA (lncRNA), pseudogenes and circular RNA don't have the ability to translate into proteins, but can act as competing endogenous RNAs (ceRNAs) to perform the post-transcriptional regulation by communicate with or co-regulate each other^11,12^. The milestone conception of ceRNA was put forward by Salmena et al. in 2011^13^. In this hypothesis, miRNA response elements (MREs) play an integral role for miRNA competitively binds to ncRNAs, by which miRNA also binds to mRNAs^13^. More generally, any RNA transcript with MREs has the potential to function as a ceRNA and repress other RNAs' activity with similar MREs. Ongoing researches have recently confirmed the role of ceRNA network in comprehensively clarifying complex gene interactions and identifying potential biomarkers for cancer diagnosis, treatment and prognosis of gastric^14^, breast^15^, pancreas^16^, bladder^17^ and HNSCC^18,19^. Fang et al. proposed a regulatory mechanism and indicated lncRNAs might have an important impact on the survival and prognosis of HNSCC patients^18^. Pan et al. comprehensively analyzed lncRNA-associated ceRNA network and identified several mRNAs and miRNAs as prognostic biomarkers of HNSCC^19^. These studies provide a better understanding how the ceRNAs contribute to improving the diagnostic and prognostic efficiency for HNSCC patients. However, the co-expression among ceRNAs has been overlooked. Weighted co-expressed gene network analysis (WGCNA) clusters highly correlated genes into one module and relates it to clinical traits, which has already been a widely-accepted tool to identify clinical biomarkers for diagnosis and therapy^20^. Previous studies have so far identified effective and reliable biomarkers for diagnosis and prognosis of HSNC patients using WGCNA approach, including CNFN^21^, APP and COL1A2^22^, which could be exploited as novel therapeutic targets for HNSCC. Consequently, we believe integral analysis of ceRNA in combination with WGCNA can be more beneficial in underlying mechanisms of cancer metastasis and prognosis, which has been rarely reported yet.

In the present study, we retrieved HNSCC-related RNA-seq data and miRNA-seq data from The Cancer Genome Atlas (TCGA) database and screened out differentially expressed lncRNAs (DElncRNAs), mRNAs (DEmRNAs), and miRNAs (DEmiRNAs) to successfully develop a ceRNA-based risk model for HNSCC patients. Though the therapeutic efficiency of HNSCC-specific prognostic signatures temporarily lacks validation by large-scale studies, our findings may provide novel insights toward developing a promising predictive tool for the metastasis and prognosis of HNSCC and lay the groundwork for further research in unveiling mechanisms of tumor progression and improving overall survival in HNSCC patients.

## Results

### Identification of DElncRNAs, DEmRNAs and DEmiRNAs

**Figure 1:**
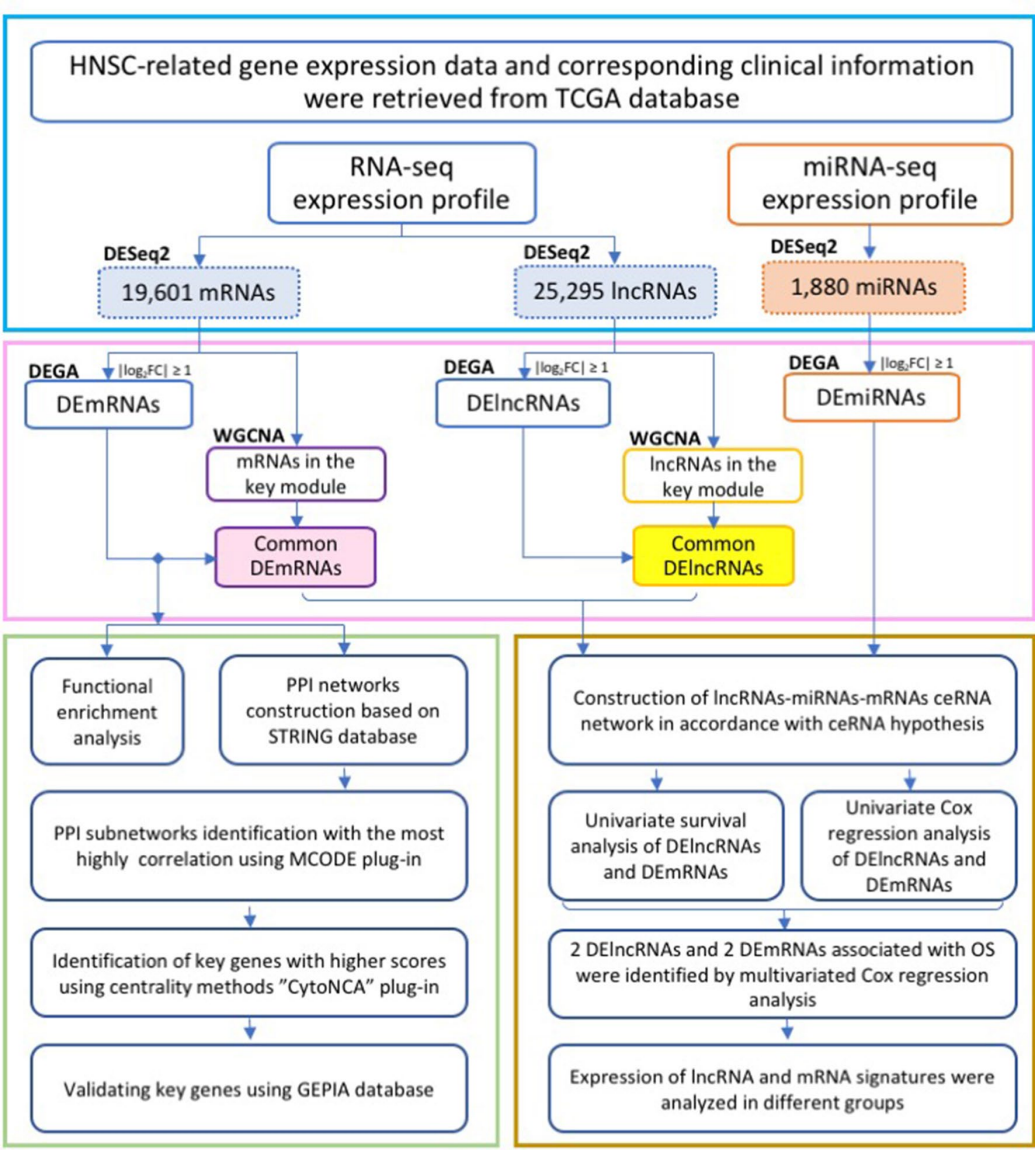
Flow chart of data preparation, processing, analysis and validation in this study.

**Figure 2:**
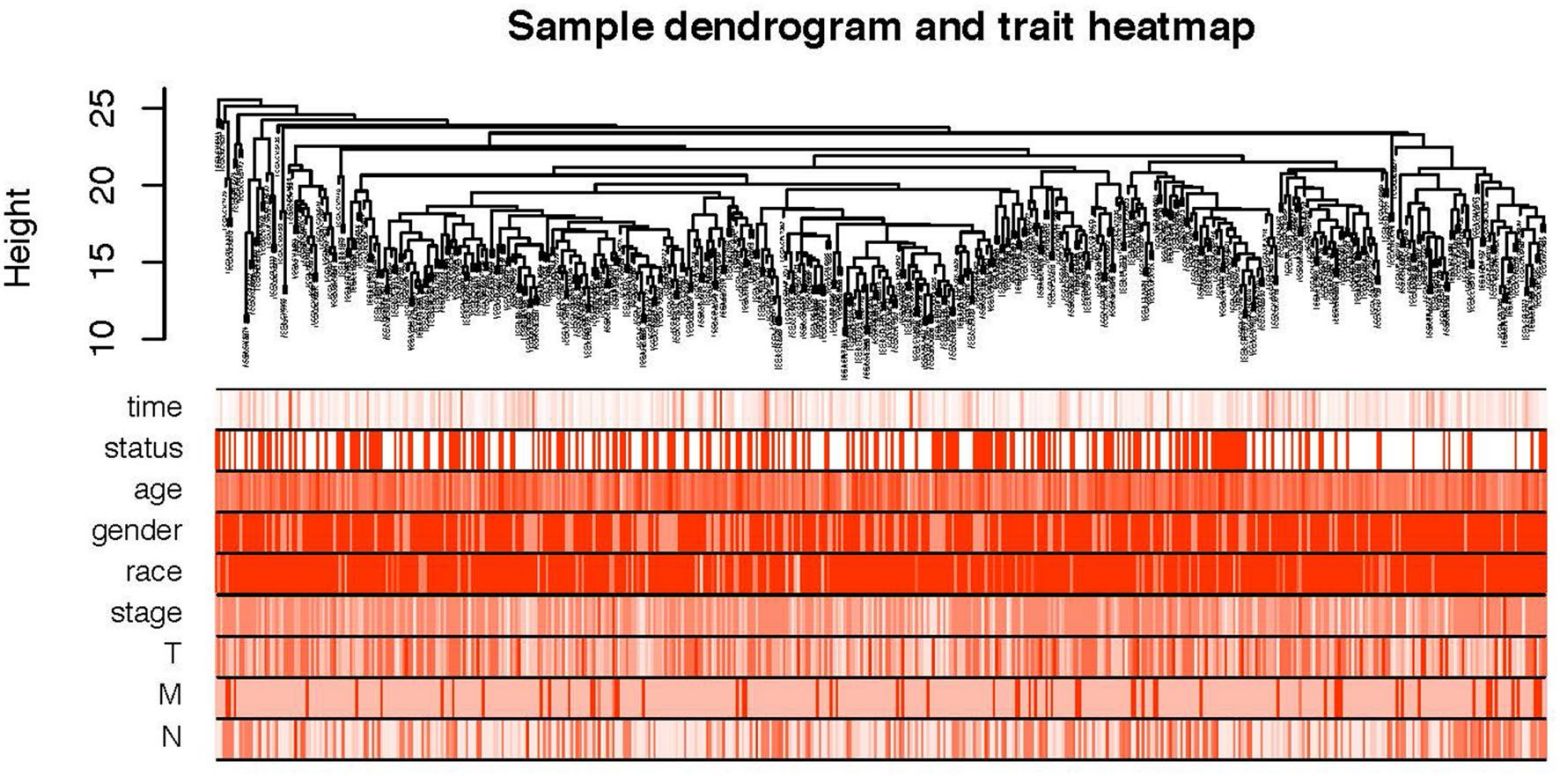
Clustering dendrogram of 502 HNSCC samples and the clinical traits. The red color represented death, female and metastasis. The color intensity was proportional to longer survival time, older age as well as higher tumor stage.

**Figure 3:**
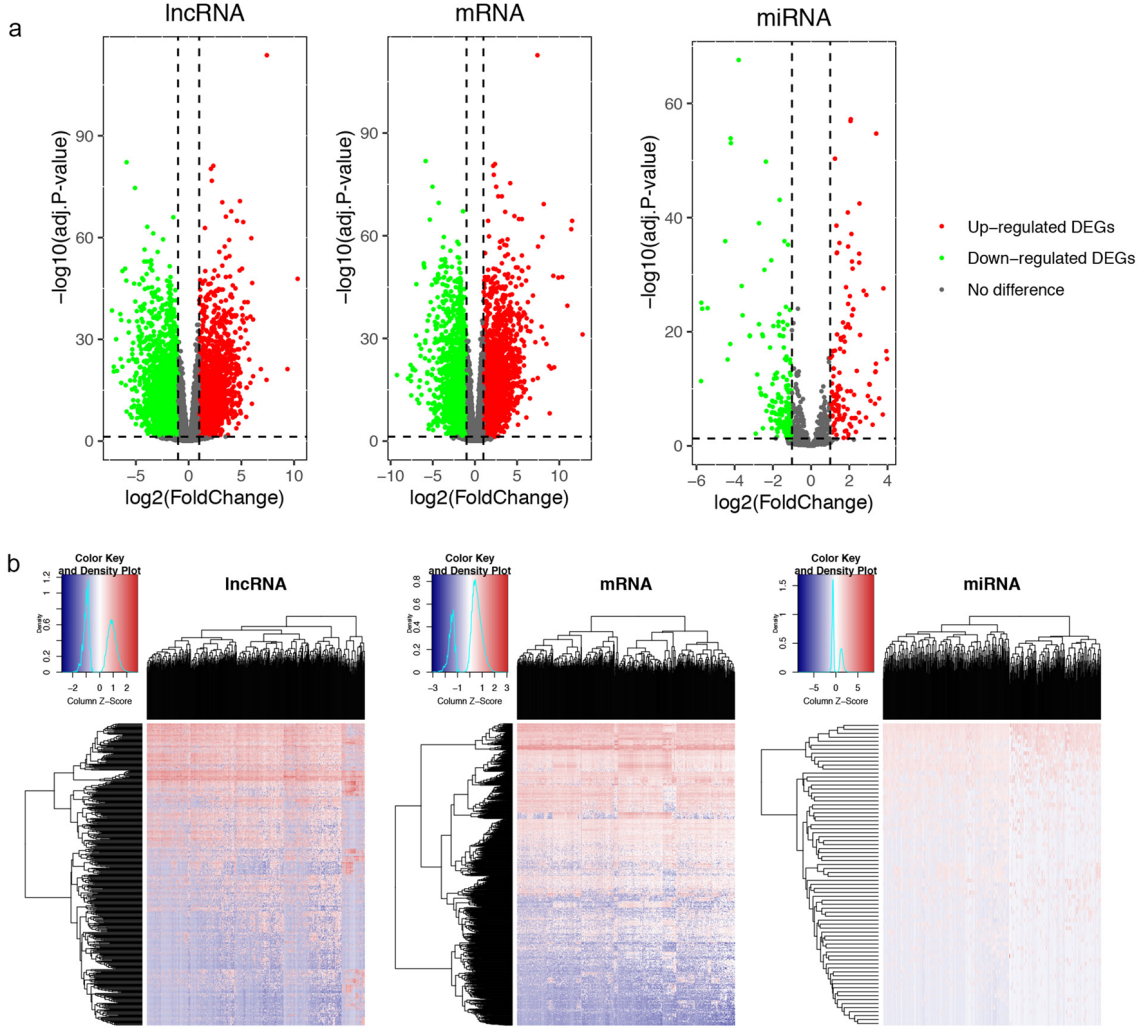
Differentially expressed genes analysis in HNSCC. (**a**) Volcano plots of expressions of lncRNAs, mRNAs and miRNAs. X axis represented the expression differences between HNSCC samples and normal samples, and Y axis represented log transformed adjusted *P* value; (**b**) heat maps of expression levels of differentially expressed lncRNAs, mRNAs and miRNAs. X axis represented HNSCC samples, and Y axis represented gene markers.

A schematic of the workflow of this work is shown in Fig. 1. The clinical features of 502 HNSCC patients were demonstrated in Fig. 2. We collected level 3 HNSCC-related RNA-seq count data of 502 HNSCC samples and 44 normal samples and level 3 miRNA-seq count data of 525 HNSCC samples and 44 normal samples from the National Cancer Institute's Genomic Data Comments data portal. In total, 25,295 lncRNAs, 19,601 mRNAs and 1880 miRNAs were extracted to implement normalization and variance-stabilizing transformation by "DESeq2" package. Using "DESeq2" package to perform differentially expressed gene analysis (DEGA), we identified DElncRNAs, DEmRNAs and DEmiRNAs between HNSCC samples and normal samples with the cut-off of |log2 (foldchange)| (|log2FC|) ≥ 1 and adjusted *P* value < 0.05. In total, 5749 DElncRNAs were screened out, including 2243 downregulated and 3506 upregulated lncRNAs in HNSCC samples. Of the 4790 DEmRNAs, 2418 were downregulated and 2372 were upregulated. Among 303 DEmiRNAs, there were 126 downregulated miRNAs and 177 upregulated miRNAs. The volcano plots displayed the distributions of DElncRNAs, DEmRNAs and DEmiRNAs (Fig. 3a) and the heat maps manifested the expression levels of these genes (Fig. 3b).

### Functional enrichment analyses of DEmRNAs

In order to shed light on biological function of DEmRNAs (|log2FC| ≥ 1, adjusted *P* value < 0.05) in HNSCC, we implemented Gene Ontology (GO) and Kyoto Encyclopedia of Genes and Genomes (KEGG) pathway analyses with a cut-off of *P* value < 0.05. 716 BPs, 140 CCs and 153 MFs were involved in GO enrichment results, and 51 KEGG pathways were significantly enriched, of which the top 10 GO terms and KEGG pathways are listed in Table 1. "Extracellular matrix" related GO terms were significantly enriching most DEmRNAs, and the most significant biological process (BP), cellular component (CC), and molecular function (MF) were Extracellular matrix organization (GO:0030198, *P* = 7.24E−22), Extracellular matrix (GO:0031012, *P* = 5.10E−23) and Extracellular matrix structural constituent (GO:0005201, *P* = 6.84E−19), respectively. Several studies have previously reported that bi-directional interactions between cancer-associated fibroblast and cancer cell depend on ECM remodeling mechanisms in HNSCC^23,24^. In the absence of natural control and balance, malignant cells are free to manipulate the ECM and eventually lead to metastasis^25^. Most DEmRNAs fell into neuroactive ligand–receptor interaction (hsa04080, *P* = 4.27E−11) pathway. Neuronal activity is regulated by neuroactive steroids that function as mediators of neurotransmitter receptors^26^. Previous researches have reported neuroactive steroids to modulate γ-aminobutyric acid (GABA) receptors that have been suggested to affect oncogenesis by controlling cell proliferation^27^. GABA_A_ receptors, one of the subclasses of GABR, are a class of transmembrane ligand-gated chloride channels, which are named based on their subunits composition: alpha (GABRA1-6), beta (GABRB1-3), gamma (GABRG1-3), delta (GABRD), epsilon (GABRE), theta (GABRQ), pi (GABRP) and rho (GABRR1-3)^28^. Among the significantly differentially expressed genes, our pathway enrichment analysis suggested that eight GABRs were involved in the neuroactive ligand–receptor interaction pathway, including GABRA4 (log2FC =  − 3.09), GABRB2 (log2FC =  − 1.09), GABRG2 (log2FC = 4.80), GABRD (log2FC = 1.86), GABRE (log2FC = 1.52), GABRP (log2FC =  − 2.49), GABRR1 (log2FC = 1.08) and GABRR2 (log2FC = 1.04).

**Table 1:** Top 10 pathways enriched by GO and KEGG analyses.

ID	Description	Adj. *P* value	Count
**Biological process (BP)**			
GO:0030198	Extracellular matrix organization	7.24E−22	159
GO:0043062	Extracellular structure organization	7.24E−22	176
GO:0006936	Muscle contraction	2.15E−17	152
GO:0003012	Muscle system process	3.97E−15	179
GO:0034765	Regulation of ion transmembrane transport	4.43E−12	172
GO:0043588	Skin development	2.09E−10	155
GO:0070252	Actin-mediated cell contraction	2.40E−10	61
GO:0030049	Muscle filament sliding	2.71E−10	30
GO:0033275	Actin-myosin filament sliding	2.71E−10	30
GO:0060047	Heart contraction	3.27E−10	113
**Cellular component (CC)**			
GO:0031012	Extracellular matrix	5.10E−23	204
GO:0043292	Contractile fiber	4.30E−19	109
GO:0062023	Collagen-containing extracellular matrix	5.36E−19	172
GO:0044449	Contractile fiber part	2.87E−18	101
GO:0030016	Myofibril	6.84E−18	102
GO:0030017	Sarcomere	6.09E−17	92
GO:0005788	Endoplasmic reticulum lumen	3.52E−15	133
GO:1902495	Transmembrane transporter complex	6.73E−15	121
GO:1990351	Transporter complex	3.28E−14	121
GO:0016324	Apical plasma membrane	3.97E−13	126
**Molecular function (MF)**			
GO:0005201	Extracellular matrix structural constituent	6.84E−19	92
GO:0022838	Substrate-specific channel activity	6.84E−19	156
GO:0022803	Passive transmembrane transporter activity	1.25E−18	166
GO:0015267	Channel activity	1.88E−18	165
GO:0030545	Receptor regulator activity	7.47E−18	181
GO:0048018	Receptor ligand activity	7.47E−18	172
GO:0005216	Ion channel activity	4.19E−17	148
GO:0008324	Cation transmembrane transporter activity	4.96E−13	186
GO:0046873	Metal ion transmembrane transporter activity	1.00E−12	140
GO:0022836	Gated channel activity	2.31E−12	117
**KEGG pathway**			
hsa04080	Neuroactive ligand–receptor interaction	4.27E−11	137
hsa05410	Hypertrophic cardiomyopathy (HCM)	5.36E−10	51
hsa04512	ECM–receptor interaction	1.71E−09	49
hsa05414	Dilated cardiomyopathy (DCM)	1.71E−09	52
hsa04974	Protein digestion and absorption	3.33E−09	51
hsa04060	Cytokine–cytokine receptor interaction	3.36E−08	113
hsa05412	Arrhythmogenic right ventricular cardiomyopathy (ARVC)	6.31E−08	42
hsa00830	Retinol metabolism	5.52E−06	35
hsa05322	Systemic lupus erythematosus	6.51E−06	57
hsa04970	Salivary secretion	1.53E−05	42

### Construction of PPI networks and validation of key DEmRNAs

**Figure 4:**
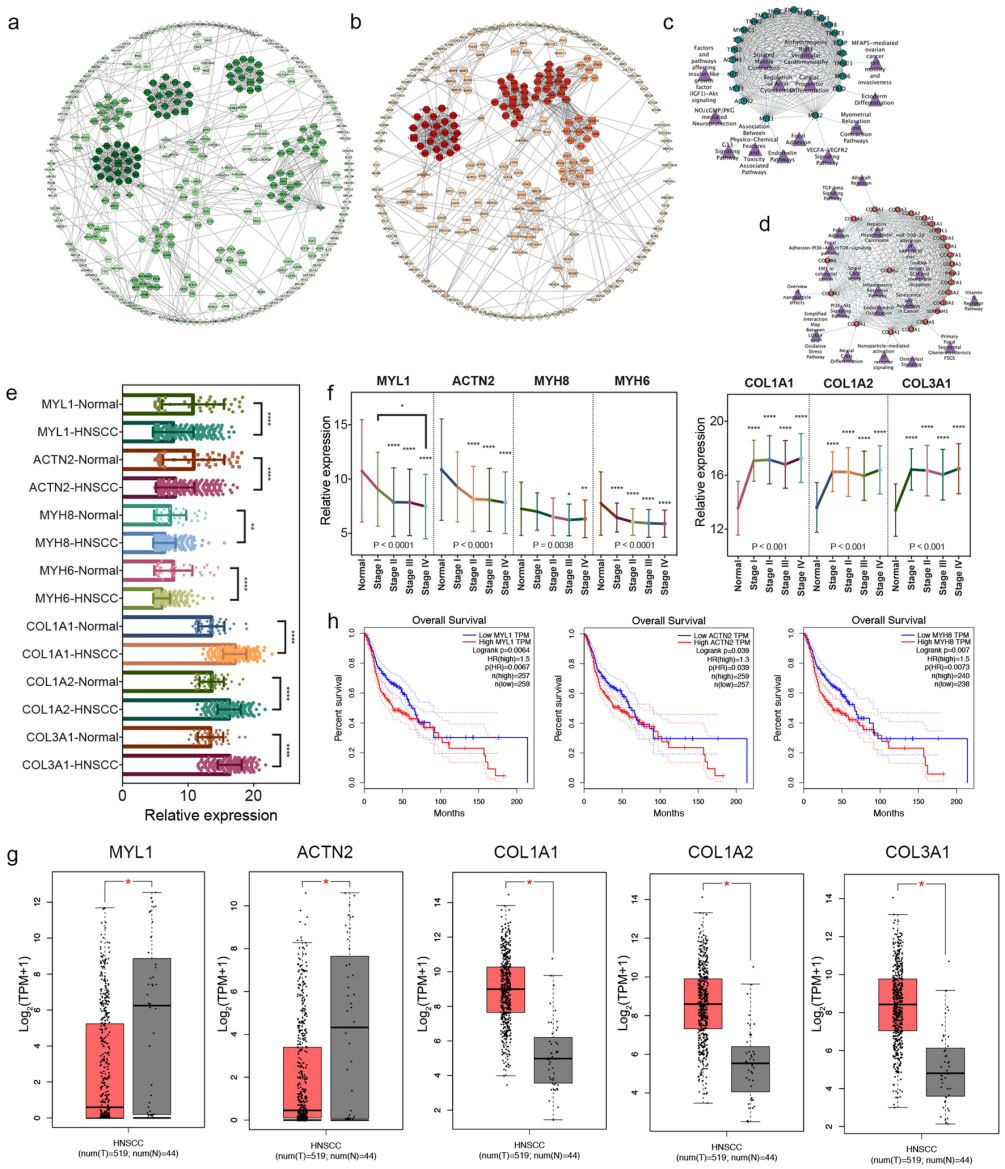
PPI networks and key mRNAs analysis in HNSCC. (**a**) Downregulated PPI network with 343 nodes and 1188 edges. The green color intensity was proportional to cluster scores. (**b**) Upregulated PPI network with 256 nodes and 1032 edges. The red color intensity was proportional to cluster scores. (**c**, **d**) Pathway-gene networks. Green circles represented downregulated DEmRNAs, and red circles represented upregulated DEmRANs. Purple triangles represented pathways. (**e**) Bar plots of expression levels of seven key mRNAs between HNSCC samples and normal samples. MYL1, ACTN2, MYH8 and MYH6 were significantly downregulated in HNSCC samples, and COL1A1, COL1A2 and COL3A1 were over-expressed. (**f**) The expressions of seven key mRNAs had significant difference between clinical stages. (**g**) Validation of gene expressions from GEPIA database. (**h**) Survival analysis. Higher expression of MYL1, ACTN2 and MYH8 resulted in worse overall survival of HNSCC patients. *: *P* < 0.05; **: *P* < 0.01; ***: *P* < 0.001; ****: *P* < 0.0001.

To select robust HNSCC-specific mRNAs, we used downregulated and upregulated DEmRNAs with |log2FC| > 2 to perform PPI analysis, and chose the gene pairs with the highest confidence (the combined score > 0.9) for further analysis, separately. In total, downregulated PPI network included 343 nodes and 1188 edges (Fig. 4a), while upregulated included 256 nodes and 1032 edges (Fig. 4b). Subnetworks selected by MCODE algorithm were displayed and clustered together based on the same cluster number, whose nodes were colored in different shades of colors according to cluster scores. The most significantly correlated module of downregulated PPI network included 22 downregulated DEmRNAs (Fig. 4c), and upregulated module covered 23 upregulated DEmRNAs (Fig. 4d). Functional pathways for each subnetwork were simultaneously established based on the "Wikipathways" database. Next, Centrality analysis of DEmRNAs in each PPI network indicated that top 1% genes obtained by the Centrality methods were MYL1 (log2FC =  − 3.92, *P* = 2.03E−9), ACTN2 (log2FC =  − 3.57, *P* = 1.10E−11), MYH8 (log2FC =  − 2.01, *P* = 0.000548), MYH6 (log2FC =  − 4.12, *P* = 1.07E−7), COL1A1 (log2FC = 2.90, *P* = 1.44E−27), COL1A2 (log2FC = 2.39, *P* = 7.76E−20) and COL3A1 (log2FC = 2.53, *P* = 7.65E−21). The seven genes were all clustered in the most significantly correlated module and considered as key DEmRNAs in HNSCC patients. Then as shown in Fig. 4e, the expression calculation based on TCGA database showed that MYL1, ACTN2, MYH8 and MYH6 were significantly downregulated in HNSCC samples, and COL1A1, COL1A2 and COL3A1 were over-expressed. In Fig. 4f, 4 downregulated mRNAs, especially MYL1, demonstrated lower expressions in advanced clinical stages when compared to normal samples. On the contrary, higher expressions of 3 upregulated mRNAs were observed in the advanced stages. Consistent with results from our analysis of the TCGA database, the subsequent expression analysis from Gene Expression Profiling and Interactive Analyses (GEPIA) database revealed that MYL1 and ACTN2 were decreasingly expressed in HNSCC patients and COL1A1, COL1A2 and COL3A1 were upregulated (Fig. 4g). Survival analysis showed that high expression of MYL1 (Log-Rank (LR) *P* = 0.0064), ACTN2 (LR *P* = 0.0039) and MYH8 (LR *P* = 0.007) presented worse OS than low expression (Fig. 4h). Finally, MYL1 and ACTN2 were confirmed their prognostic association with HNSCC in TCGA and GEPIA database, respectively, and were subsequently included in further analysis.

### Identification of co-expression module by WGCNA

**Figure 5:**
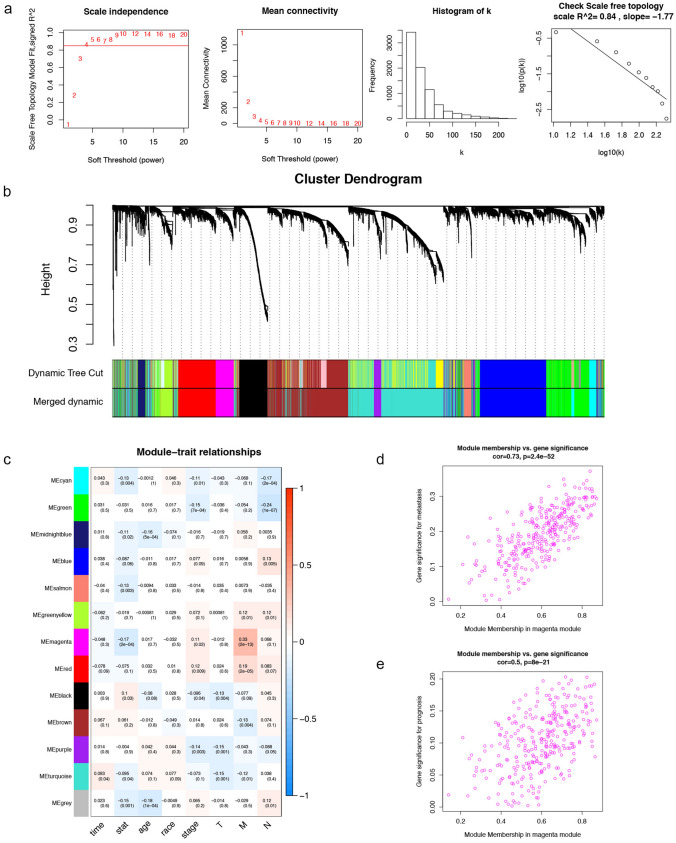
Co-expression network analysis by WGNCA. (**a**) Soft-threshold power analysis for network topology and the test of property of scale-free network. (**b**) Dendrogram of all DEmRNAs clustered based on a dissimilarity measure (1-TOM). (**c**) The relationships between modules and clinical traits. Magenta module was identified to be the most significant association with metastasis and prognosis in HNSCC patients. (**d**) The correlation between the magenta module membership and metastasis. (**e**) The correlation between the magenta module membership and prognosis.

19,601 mRNAs and 25,295 lncRNAs retrieved from TCGA RNA-seq data were used to establish co-expression networks, respectively. 489 HNSCC patients with complete clinical information were selected to be analyzed using WGCNA algorithm. Pearson correlation matrix among mRNAs were converted into an adjacency matrix that was strengthened by the best power (β = 4) with scale-free topology criterion of R^2^ = 0.84 (Fig. 5a). Through minimizing interference from noise and spurious associations by topological overlap measure (TOM), mRNAs with identical expression profiles were grouped together into gene modules. Then, gene modules with 70% similarity were merged into one module by dynamic cutting algorithm (Fig. 5b). From this analysis, 12 gene modules were identified for downstream analysis. Module-trait relationship (MTR) analysis results showed that the magenta module was found to have the highest association with metastasis (*P* = 2E−13) and prognosis (*P* = 0.00002) in HNSCC patients (Fig. 5c). The module membership in the magenta module (including 307 mRNAs) suggested the correlation with gene significance (GS) was 0.73 (*P* = 2.4E−52) for metastasis (Fig. 5d) and 0.5 (*P* = 8E−21) for prognosis (Fig. 5e). Parallel processing and analysis were synchronously implemented among lncRNAs, of which the results were showed in Supplementary Figure 1. The yellow module was most significantly correlated with metastasis (*P* = 1E−14) and prognosis (*P* = 0.00003), including 743 lncRNAs. The metastatic correlation between the module membership of the yellow module and GS of 743 lncRNAs was 0.82 with *P* = 9.7E−182, and the prognostic correlation was 0.61 with *P* = 6.3E−77. Finally, the magenta module in mRNAs co-expression network and the yellow module in lncRNAs co-expression network were selected for further analysis.

### Construction of the ceRNA networks

**Figure 6:**
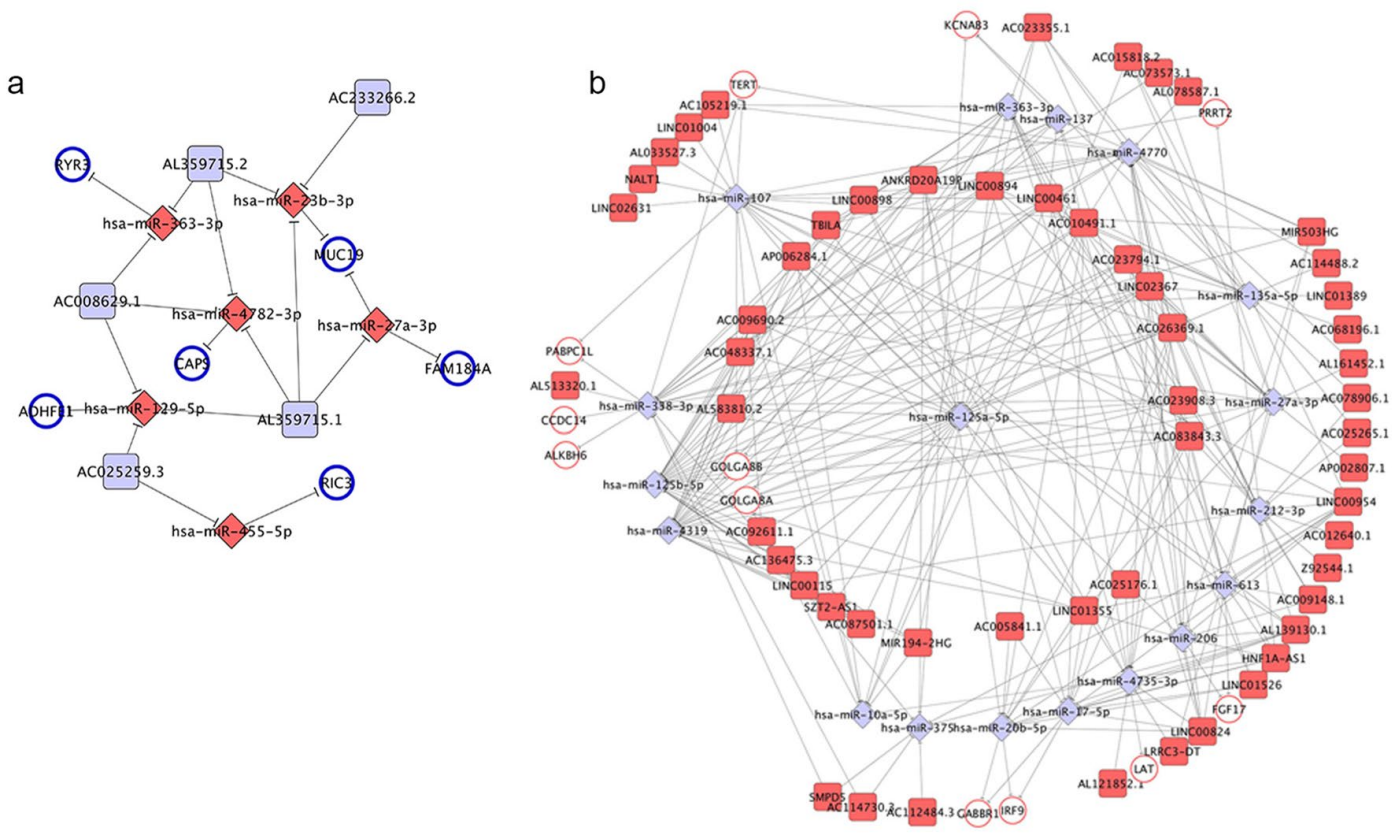
LncRNA-miRNA-mRNA ceRNA network. (**a**) Under-expressed network. (**b**) Over-expressed network. Blue and red color nodes represented downregulated and upregulated RNAs, respectively. Circle represented mRNA, and rhombus represented miRNA and round rectangle represented lncRNA.

According to the results of WGCNA and DEGA, 67 out of 307 mRNAs in the magenta module and 241 out of 743 lncRNAs in the yellow module were differentially expressed in HNSCC patients. Then 67 DEmRNAs (17 downregulated and 50 upregulated), 241 DElncRNAs (33 downregulated and 208 upregulated) and 303 DEmiRNAs (126 downregulated and 177 upregulated) were required to construct lncRNA-miRNA-mRNA ceRNA networks. Dependent upon the hypothesis of ceRNA and expression levels of DERNAs, ceRNA networks divided into two types: under-expressed and over-expressed networks. In all, the under-expressed ceRNA network included 5 downregulated DElncRNAs, 6 downregulated DEmRNAs and 6 miRNAs (1 upregulated DEmiRNAs) and in combination with 20 edges (Fig. 6a). In the over-expressed ceRNA network, 55 upregulated DElncRNAs and 12 upregulated DEmRNAs were connected by 18 common miRNAs (5 downregulated DEmiRNAs) and 282 edges were involved (Fig. 6b). Among the 6 downregulated DEmRNAs and 12 upregulated DEmRNAs, we found that GABBR1 was involved in the neuroactive ligand–receptor interaction pathway and RYR3 was associated with ion channel activity.

### Survival analysis of lncRNA and mRNA signatures

489 HNSCC patients were divided into high- and low-expression groups according to the median value of gene expression level. The association of overall survival (OS) in HNSCC patients and DElncRNAs and DEmRNAs in ceRNA networks were evaluated by Kaplan–Meier (KM) curve analysis and LR test. Low expression of 11 lncRNAs and 6 mRNAs predicted poorer OS than high expression in HNSCC patients (Supplementary Figure 2). Among them, 1 out of 11 lncRNAs and 4 out of 6 mRNAs were downregulated, while the others were upregulated in HNSCC patients.

### Establishment of HNSCC-specific prognostic model for lncRNA and mRNA signatures

**Figure 7:**
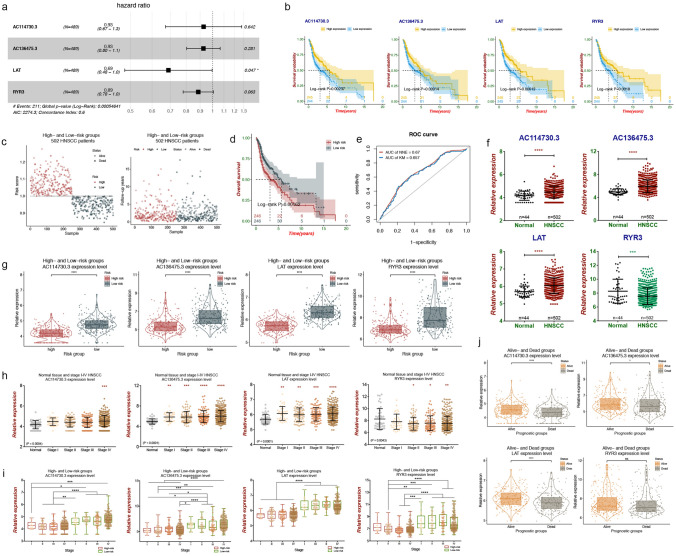
HNSCC-specific prognostic risk model and validation. (**a**) The prognostic model including 2 lncRNAs and 2 mRNAs. (**b**) Kaplan–Meier survival curves for 2 lncRNAs and 2 mRNAs associated with OS of HNSCC patients by Log-rank test. X axis represented overall survival time (years) and Y axis represented survival probability. (**c**) The HNSCC patients were divided into high- and low-risk groups based on the risk scores of HNSCC patients. (**d**) Survival analysis suggested high risk group had worse prognosis in HNSCC patients. (**e**) The ROC analysis showed the AUC of the prognostic model was 0.67 and 0.657 that were calculated by NNE and KM method, respectively. (**f**) Expression patterns of the 2 lncRNAs and 2 mRNAs between HNSCC samples and normal samples. (**g**) Violin plots of expression patterns between high-risk group and low-risk group. (**h**) Comparison of expression levels for 2 lncRNAs and 2 mRNAs between normal samples and stage I, II, III, and IV HNSCC samples. (**i**) Comparison of expression levels for 2 lncRNAs and 2 mRNAs at different clinical stages between high-risk and low-risk groups. (**j**) Violin plots of expression patterns between alive and dead patients. *: *P* < 0.05; **: *P* < 0.01; ***: *P* < 0.001; ****: *P* < 0.0001.

Using the univariate regression analysis, we identified potential lncRNAs and mRNAs from ceRNA networks with prognostic values for HNSCC. Among them, 10 lncRNAs and 10 mRNAs were detected to be significantly associated with OS of HNSCC (Table 2). Meanwhile, we found that 6 lncRNAs (AC009148.1, AC073573.1, AC114730.3, AC136475.3, AL139130.1 and AL513320.1) and 6 mRNAs (CAPS, MUC19, RIC3, RYR3, LAT and PRRT2) were simultaneously identified to have prognostic values in LR test and univariate Cox regression analysis. Subsequently, in order to establish HNSCC-specific prognostic model, the multivariate Cox regression analysis were performed among the lncRNAs (AC114730.3 and AC136475.3) and mRNAs (LAT, RYR3) with stronger correlation to OS (*P* < 0.01). As shown in Fig. 7a, the prognostic model was established (*P* = 0.00055). High lncRNA expression of AC114730.3 and AC136375.3, and high mRNA expression of LAT and RYR3 contributed to better OS in HNSCC patients (Fig. 7b). Based on the median value of risk score for all patients, HNSCC patients were divided into high- and low-risk groups (Fig. 7c). In the high-risk group, the mortality rate was significantly higher (*P* = 0.0016) and the prognosis was worse when compared to the low-risk group (Fig. 7d). Furthermore, in Fig. 7e, the area under curve (AUC) of the prognostic model was 0.67 and 0.657 that were calculated by NNE and KM method, respectively, and which indicated the 2 lncRNAs and 2 mRNAs could be effective prognostic biomarkers in predicting OS for HNSCC patients.

**Table 2:** Univariate Cox proportional hazards regression analysis for DElncRNAs and DEmRNAs in ceRNA networks.

ID	Gene name	Beta	HR (95% CI)	*P* value
**lncRNAs**				
ENSG00000261326	LINC01355	− 0.21	0.81 (0.71–0.93)	0.0029
ENSG00000235351	AC114730.3	− 0.38	0.69 (0.53–0.89)	0.0053
ENSG00000255026	AC136475.3	− 0.17	0.84 (0.74–0.96)	0.0086
ENSG00000251455	AC092611.1	− 0.29	0.75 (0.59–0.95)	0.017
ENSG00000257808	AC073573.1	− 0.48	0.62 (0.41–0.94)	0.024
ENSG00000260495	AC009148.1	− 0.28	0.75 (0.59–0.97)	0.025
ENSG00000237390	AL139130.1	− 0.46	0.63 (0.42–0.94)	0.025
ENSG00000238260	AL513320.1	− 0.31	0.73 (0.56–0.97)	0.028
ENSG00000235790	AC114488.2	− 0.26	0.77 (0.61–0.98)	0.034
ENSG00000261360	AC010491.1	− 0.29	0.75 (0.57–0.99)	0.042
**mRNAs**				
ENSG00000213658	LAT	− 0.57	0.57 (0.42–0.77)	0.00027
ENSG00000170049	KCNAB3	− 0.34	0.71 (0.56–0.89)	0.0033
ENSG00000198838	RYR3	− 0.17	0.84 (0.74–0.95)	0.0065
ENSG00000164362	TERT	− 0.28	0.76 (0.61–0.93)	0.0081
ENSG00000167371	PRRT2	− 0.28	0.75 (0.59–0.96)	0.02
ENSG00000105519	CAPS	− 0.16	0.85 (0.74–0.98)	0.023
ENSG00000239382	ALKBH6	− 0.27	0.77 (0.61–0.97)	0.024
ENSG00000205592	MUC19	− 0.38	0.69 (0.49–0.96)	0.026
ENSG00000166405	RIC3	− 0.18	0.83 (0.71–0.98)	0.026
ENSG00000158815	FGF17	− 0.31	0.73 (0.54–0.99)	0.041

To further confirm the metastatic and prognostic roles of the lncRNAs and mRNAs, we analyzed their expression levels between different groups. Firstly, the expression levels between HNSCC samples and normal samples were evaluated, and the results showed AC114730.3, AC136375.3 and LAT were significantly over-expressed in HNSCC patients, whereas RYR3 was downregulated (Fig. 7f). When compared to the low-risk group, all the four RNAs were significantly decreased expression in the high-risk group, demonstrating higher risk scores (Fig. 7g). Furthermore, we evaluated their expression levels when took the clinical stages into account (Fig. 7h). AC136475.3 and LAT obviously over-expressed in stage I, II, III, and IV HNSCC samples compared with normal samples, respectively. AC114730.3 expressed higher only in the stage IV than in normal samples. RYR3 showed lower expression level except stage I. It's surprising that gene expression levels in the high-risk group were lower than that in the low-risk group at different clinical stages, which suggested some of the patients with clinical early-stage (stage I or II) were supposed to belong to the high-risk group and were likely to have a poor prognosis (Fig. 7i). Moreover, the expressions of the RNAs (expect RYR3) between alive- and dead-group were also observed, which were consistent with the above expression patterns (Fig. 7j).

### Verification of MYL1, ACTN2 and LAT protein expression levels

**Figure 8:**
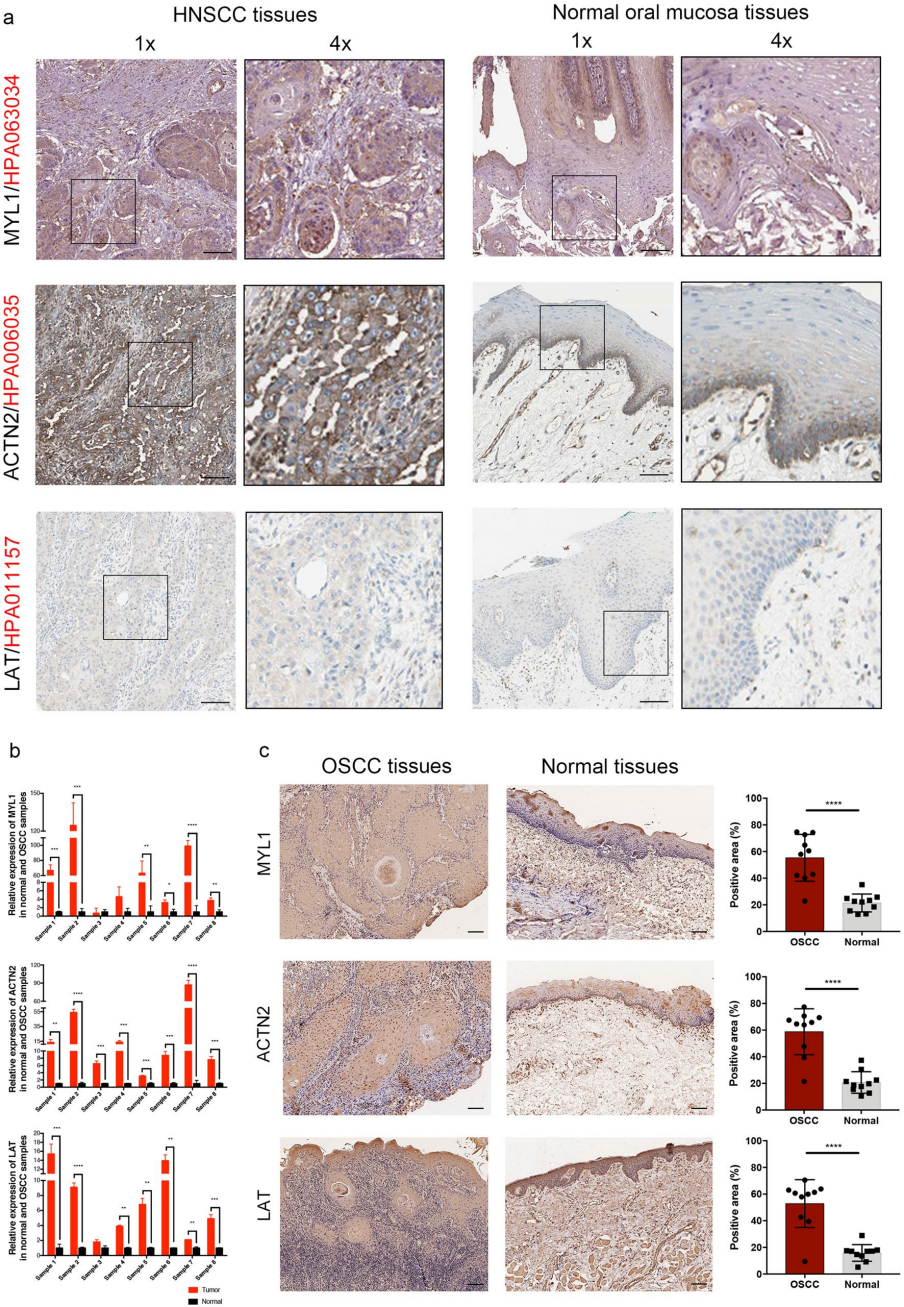
MYL1, ACTN2 and LAT expression validation. (**a**) Protein expression levels in HNSCC as compared to those in normal tissues by IHC staining from the Human Protein Atlas database. Black square boxes represent typical normal and HNSCC tissues, respectively. (**b**) Comparison of gene expression level by using qPCR. (**c**) IHC staining of the proteins in OSCC tissues and normal oral mucosa tissues. Scale bars represent 100 µm. *: *P* < 0.05; **: *P* < 0.01; ***: *P* < 0.001; ****: *P* < 0.0001.

To explore the protein expression patterns of the prognosis related genes in HNSCC samples, we examined the expression of MYL1, ACTN2 and LAT in The Human Protein Atlas database (Fig. 8a). RYR3 protein expression level was not evaluated because its information was not available in the database. Besides, cancerous and para-cancerous tissues from oral squamous cell carcinoma (OSCC) patients were collected from The Affiliated Hospital of Stomatology, Zhejiang University School of Medicine. Verification of gene expression levels and protein expression levels were demonstrated in Fig. 8b,c. Consistent with our bioinformatic analysis results, the protein expression patterns demonstrated that MYL1, ACTN2 and LAT were strongly over expressed in OSCC tissues than in normal oral mucosa tissues. IHC staining revealed the cytoplasmic membranous location of the MYL1, ACTN2 and LAT proteins in cancerous tissues. Atypia of cancerous cell was shown in the nests. The boundary between epithelial tissue and connective tissue becomes blurred in cancerous tissues, which was clearly well-structured in the normal oral mucosa tissues. All patients' information is provided in Tables 3 and 4.

**Table 3:** IHC staining characteristics of genes from The Human Protein Atlas database.

Gene	Patient ID	Gender	Age	Staining	Intensity	Quantity	Location
**Normal oral mucosa tissue**							
MYL1	5345	Male	9	Low	Weak	75–25%	Cytoplasmic membranous
ACTN2	1711	Male	71	Low	Moderate	< 25%	Cytoplasmic membranous
LAT	3135	Male	58	Not detected	Negative	None	None
**Head and neck squamous cell carcinoma tissue**							
MYL1	5286	Male	56	Medium	Moderate	> 75%	Cytoplasmic membranous
ACTN2	2624	Male	87	High	Strong	> 75%	Cytoplasmic membranous
LAT	2608	Male	51	Low	Weak	75–25%	Cytoplasmic membranous

*IHC* immunohistochemistry.

**Table 4:** Histopathological characteristics of the OSCC patients.

Order	Patient ID	Gender	Age (years)	Location	Tumor size (cm^3^)	Differentiation^#^
1	00013232	Male	64	Gum	3.8 * 2.0 * 1.8	High
2	00013195	Male	76	Lip	3.0 * 2.0 * 0.7	High
3	00013006	Female	64	Tongue	2.5 * 1.5 * 0.9	Medium–high
4	00012796	Male	49	Tongue	3.8 * 1.6 * 1.0	Medium–high
5	00012792	Male	57	Tongue	1.0 * 1.0 * 0.2	Medium–low
6	00012679	Female	45	Tongue	4.5 * 2.2 * 1.0	Medium–high
7	00012380	Male	55	Tongue	1.5 * 1.0 * 0.8	Medium–high
8	00001858	Male	75	Gum	1.2 * 1.0 * 0.5	Medium–low
9	00011717	Female	58	Lip	1.5 * 1.0 * 0.9	High
10	00010176	Female	53	Gum	2.7 * 1.8 * 0.7	Low

*IHC* immunohistochemistry.

^#^Diagnosed by two experienced pathologists.

## Discussion

HNSCC is a common malignant cancer with high genetic diversities, and per year causes hundreds of thousands of physical dysfunctions and aesthetic problems, even deaths^1^. Especially, advanced HNSCC with high metastatic rate usually suggests poor overall survival because conventional therapeutic strategies have achieved unsatisfactory results^2^. In order to promote outcomes of this disease, novel therapeutic targets with higher specificity and efficacy are urgently needed, which are also expected to be potential predicting biomarkers for HNSCC. It's well documented that lncRNAs play chief roles in many biological processes by ceRNA mechanism, such as tumorigenesis and progression. In this study, we systematically analyzed the HNSCC-related RNA-seq and miRNA-seq data from TCGA database by integrated bioinformatics analyses including GO and KEGG pathway analyses, PPI network construction, WGCNA, ceRNA network establishment and survival analysis, and screened out the HNSCC-specific prognostic lncRNA and mRNA signatures to establish a novel ceRNA-based risk model. Finally, four mRNAs (MYL1, ACTN2, LAT and RYR3) and two lncRNAs (AC114730.3 and AC136375.3) were identified to associate with OS in HNSCC patients.

MYL1 (myosin light chain 1) encodes an alkali light chain for myosin that is a hexametric ATPase cellular motor protein. It was well studied that MYL1 played a key role in congenital myopathy and knockdown of the gene could cause severe muscle-related disorders in zebrafish^29^. Recently, MYL1 has been identified as a potential biomarker in various cancers^30,31^. Sajnani et al. found that MYL1 was experimentally proved to be downregulated in buccal cancer and be involved in the "actin mediated cell contraction" biological progress^31^, which was consistent with our results. ACTN2 (actin alpha 2) is a member of the spectrin gene superfamily that includes varying groups of cytoskeletal proteins. In breast cancer patients, mutated ACTN2 was found to be related to invasive ductal carcinoma and suggested a worse OS than ductal carcinoma in situ^32^. Sun et al. and his colleagues reported ACTN2 was one of the hub genes selected by bioinformatics methods in PTEN mutation prostate cancer^33^. However, so far researches on the associations between aberrantly expressed ACTN2 and tumorigenesis and progression of HNSCC are barely reported. The current study firstly indicated negative MYL1 and ACTN2 expression contributed to a better OS of HNSCC patients. Perhaps the low expression of the genes was to inhibit some activities in the tumor cells, so patients with lower expression of these genes showed better prognosis.

With the development of bioinformatics analysis methodology, the single method to find a limited group of genes as therapeutic targets has been less convincing, so the comprehensive bioinformatics analyses are more recognized by researchers. The ceRNA theory points out that lncRNAs could function as ceRNAs and competitively bind to miRNAs by acting as "sponges" of miRNAs, which might derepress activity or expression of those miRNA-mediated target with similar binding sites^34^. This analysis may shed light on regulatory networks that have been underestimated by conventional protein-coding studies. Heretofore, some studies have investigated ceRNAs in HNSCC and identified several RNAs associated with OS of HNSCC patients^35,36^. For instance, Gao et al. comprehensively investigated TCGA data to find HOXC13-AS was highly expressed in HNSCC, and further confirmed knockdown of HOXC13-AS impaired proliferation, migration and invasion of nasopharyngeal carcinoma cell via targeting miR-383-3p/HMGA2 axis^35^. Lately, Cai et al. revealed the prognostic roles of miRNA-204/211 in HNSCC by integrally analyzing sequencing data from TCGA database to construct ceRNA networks^36^. These studies provide a better understanding how the ceRNA contributes to improving the diagnostic and prognostic efficiency of HNSCC patients. However, rare studies have focused on the co-expression among ceRNAs, let alone establishing risk models. In the present study, we innovatively used WGCNA to identify co-expressed modules in HNSCC, and further constructed lncRNA-miRNA-mRNA ceRNA network based on the co-expressed genes in the most significant module. Moreover, LR test and univariate Cox regression analysis selected 12 RNAs (including 6 lncRNAs and 6 mRNAs) that were simultaneously having significant prognostic values in HNSCC patients. LAT, RYR3, AC114730.3 and AC136375.3 were ulteriorly implemented multivariate Cox regression analysis to build a HNSCC-specific prognostic model, since their stronger correlations to OS of HNSCC.

The protein encoded by LAT (linker for activation of T cells) can recruit multiple downstream molecules after phosphorylation and activation, and is involved in immunology related pathways^37^. T cell is an important component of acquired immunity for hosts, which is able to be activated by phosphorylated LAT through TCR signaling pathway^38^. Wang et al. elucidated prognostic risk models containing 3 genes by comprehensively analyzing methylation data and RNA-seq data for clear cell renal cell carcinoma (ccRCC) from TCGA database, and concluded that hypomethylation and over-expression of LAT resulted in poor OS of ccRCC^39^. Nevertheless, no studies so far have reported any correlation between LAT and HNSCC. In our study, we found that LAT was highly expressed in HNSCC patients and correlated with tumor metastasis and prognosis, and those patients with higher expression of LAT surprisingly presented lower risk scores and better survival. According to our findings and previous literatures, we speculate that LAT, as a protective gene, takes a good effect on preventing tumor metastasis and deterioration in HNSCC. On the contrary, RYR3 (ryanodine receptor 3), one of the isoforms of RYRs, encodes protein to release calcium from intracellular storage^40^. The present study demonstrated that RYR3 was apparently downregulated in HNSCC tissues when compared to normal samples, and lower expression of RYR3 always led to higher risk scores and worse OS in HNSCC patients. However, none of the previous studies has been elucidated its biological role in HNSCC. Schmitt K et al. held that reduced expressed RYR2, another isoform of RYRs, might serve as risk factor for unfavorable prognosis and upcoming malignant conversion in head and neck cancer^41^. Using siRNA or miR-367 to knockdown the endogenous RYR3 apparently inhibited growth and migration of breast cancer cells, which led cell–cell contacts became more weakened because of their rounder morphology^42^. Therefore, tumor suppressor role of RYR3 on HNSCC still need to be further clarified.

Between the identified 2 lncRNAs in the risk model, AC114730.3 and AC136375.3 were remarkably over-expressed in HNSCC tissues, and HNSCC patients with higher expressions presented lower risk scores and better survival, which were similar to LAT expression pattern. Although no studies so far have reported any correlation between the 2 lncRNAs and cancer, our study firstly detected the upregulated AC114730.3 in ceRNA network competed with downregulated miR-375 and miR-338-3p to indirectly regulate downstream mRNAs. Dependent upon various cancer types, miR-375 tended to have dramatically suppressive or oncogenic effect on cancers^43–45^. MiR-375 could inhibit the growth and metastasis of ovarian cancer cells, of which the suppressive effect was reversed by MLK7-AS1 lncRNA that indirectly upregulated YAP1 expression^43^. The MLK7-AS1 regulatory role towards miR-375 was observed in gastric cancer as well^44^. However, lower miR-375 expression level was evaluated in malignant basal-like breast cancer than in luminal-like breast cancer, suggesting malignant development of tumor^45^. The current study found AC114730.3 was aberrantly expressed in HNSCC and functioned as a novel regulatory lncRNA of miR-375, which triggered our speculation that over-expressed AC114730.3 inhibited miR-375 expression, but might as similar to breast cancer, the miR-375 would tend to act as a tumorigenesis molecular in HNSCC. Besides, among the potential targeting mRNAs for miR-375, KCNAB3 was surprisingly upregulated in HNSCC, composing AC114730.3/miR-375/KCNAB3 regulatory axis. Thus, it is reasonable to understand why highly expressed AC114730.3 contributed to better OS in HNSCC patients. In addition, miR-338-3p was predicted to be another targeting miRNA of AC114730.3 and no researches have so far focused on its effect on HNSCC patients. Not coincidentally, such a miRNA deserves our attention since its reported association with non-small cell lung cancer (NSCLC)^46^, cervical cancer^47^ and glioma^48^. Yang et al. found over-expressed LINC00525 suggested poor prognosis and could modulate miR-338-3p that endogenously targeted IRS2 in NSCLC^46^. Two circular RNA, HIPK3 and SMO, were reported to promote tumor proliferation and metastasis through sponging miR-338-3p in cervical cancer^47^ and glioma^48^, respectively. Based on our findings and published literatures, however, we are weak in understanding and explaining the mechanisms of AC114730.3 and miR-338-3p about their prognostic effect on HNSCC. In brief, findings in the current study indicate that MYL1, ACTN2, LAT, AC114730.3 and AC136375.3 may play roles as HNSCC suppressors, while RYR3 may function as a HNSCC promoter.

Although ceRNA interactions have been described by multiple studies, the molecular conditions for optimal activity of ceRNA remain ambiguous^12^. The ceRNAs were required to compete with the entire target pool of miRNAs to bind targets. In order to win the "one versus multiple" battle, the abundance of ceRNA must to be increased to an aberrantly high level. A previous study has revealed up to 23-fold increase of ALDOA abundance did not lead to detectable miR-122 inhibition in hepatocytes^49^. Except for the abundance of ceRNA, other factors might also have profound effects on ceRNA crosstalk, including localization of ceRNA^13^, binding affinity of the shared MREs^13^, secondary structure of RNA^50^, RNA editing^51^ and so on. Therefore, current ceRNA research is still in its infancy so that one of the exciting avenues for future work is to uncover the mystery of their widespread crosstalk. Some limitations must be noted despite the construction the risk model in clinical implication. Firstly, the ceRNA-based risk model must be verified by experimental approaches to further elucidate the precise molecular mechanisms underlying HNSCC. Secondly, the predictive and therapeutic efficiency of HNSCC-specific prognostic signatures needs to be evaluated by large-scale studies.

In conclusion, four mRNAs and two lncRNAs were identified as HNSCC-specific prognostic signatures for HNSCC patients. Moreover, gene functions, co-expressed modules and ceRNA-based prognostic risk model were also elucidated, which facilitated the expansion of the current study on the roles of ceRNAs in the tumorigenesis, development and treatment of head and neck squamous cell carcinoma. Further experimental studies are required to biologically validate these findings.

## Materials and methods

### Data acquisition

HNSCC-related RNA-sequencing (RNA-seq) data and miRNA-sequencing (miRNA-seq) data that were derived from the IlluminaHiSeq_RNASeq and IlluminaHiSeq_miRNASeq sequencing platforms, were retrieved from TCGA database (https://www.cancer.gov/tcga), respectively. The RNA and miRNA expression profiles (level 3) were free to downloaded from the National Cancer Institute's Genomic Data Comments data portal (https://portal.gdc.cancer.gov). The RNA-seq data included 502 HNSCC samples and 44 normal samples, while the miRNA-seq data included 525 HNSCC samples and 44 normal samples. Among them, 44 normal samples of RNA-seq and miRNA-seq data were from normal tissues nearby tumorous tissues of 44 HNSCC patients. We also simultaneously downloaded detailed clinical and follow-up information of all HNSCC patients from TCGA database. Patients who met the following inclusion criteria were involved in TCGA-HNSCC cohort: (1) histologically diagnosed HNSCC; (2) no other malignancy except HNSCC; (3) patients with detailed clinical and follow-up information.

### RNA-seq and miRNA-seq data preprocessing and differentially expressed gene analysis (DEGA)

Subsequently, mRNAs and lncRNAs were annotated based on GENCODE Release 35 (GRCh38.p13) (https://www.gencodegenes.org/human/) and miRNAs were encoded according to miRbase v22 (http://www.mirbase.org/index.shtml#opennewwindow). Level 3 RNA-seq and miRNA-seq count data were normalized by size factor^52^ and transformed via variance-stabilizing transformation (VST) implemented using "DESeq2" package^53^. This transformation makes the expression values homoscedastic by fitting the dispersion to a negative binomial distribution. "DESeq2" package in Bioconductor project (version 3.10, http://www.bioconductor.org) was utilized to perform DEGA. Then, we screened out DElncRNAs, DEmRNAs and DEmiRNAs between HNSCC samples and normal samples. |Log2FC| ≥ 1 and adjusted *P* value < 0.05 were set as cut-off criteria. The volcano plots of lncRNA, mRNA and miRNA expression were constructed using the "ggplot2" package and the heat maps of DElncRNAs, DEmRNAs and DEmiRNAs were plotted using the "pheatmap" package in R software (version 3.6.0)^54^.

### Functional enrichment analyses of DEmRNAs

We next implemented functional enrichment analyses so as to shed light on biological function of DEmRNAs. To do this, GO and KEGG pathway^55^ analyses of all DEmRNAs was conducted using the "clusterProfiler" package^56^ in R software. For this analysis, the statistical cutoff threshold was set at adjusted *P* value < 0.05. GO analysis included three categories: BP, CC, and MF.

### PPI network construction and analysis of DEmRNAs

In accordance with expression levels, DEmRNAs were separated into two groups and then separately used to build PPI networks. DEmRNAs with cut-off of |log2FC| ≥ 2 and adjusted *P* value < 0.05 were uploaded to the Search Tool for the Retrieval of Interacting Genes (STRING, version 11, https://string-db.org) database. The gene pairs with a combined score ≥ 0.9 (the highest confidence) were retrieved from the results and visualized in Cytoscape software (version 3.7.0). Next, the highly correlated module was screened out from whole PPI network using the MCODE plugin in Cytoscape software to perform Molecular Complex Detection algorithm. The most significantly correlated module of each group was selected for further investigation. Then, we performed functional pathway enrichment analysis among clustered DEmRNAs in the most significantly correlated module based on the "Wikipathways" database using CyTargetLinker plugin in Cytoscape software.

### Identification of key DEmRNAs and validation

Another plugin in Cytoscape software, CytoNCA, was used to perform Centrality analyses that included Subgraph Centrality, Degree Centrality, Eigenvector Centrality, Information Centrality, Betweenness Centrality, Closeness Centrality and Network Centrlity^57^. Key DEmRNAs in each PPI network were identified by Centrality analyses. We selected the DEmRNAs with top 1% Centrality scores as key DEmRNAs. Next, we calculated expression levels of key DEmRNAs between HNSCC samples and normal samples, and further evaluated the relationship between HNSCC samples and normal samples in different stages. Moreover, GEPIA (http://gepia.cancer-pku.cn) database has been previously and publicly adopted to analyze gene expression levels of normal and tumorous samples from TCGA. Thus, we also performed the expression analysis between normal and HNSCC samples using log2(TPM + 1) in GEPIA database. Besides, survival analysis of key DEmRNAs was implemented using Log-Rank test. The *P* value, hazard ratio and the 95% confidence interval were calculated as well.

### Weighted gene co-expression network analysis

In order to construct the co-expression network and identify co-expression gene modules, we performed WGCNA among lncRNAs and mRNAs retrieved from the TCGA RNA-seq data, respectively. After removing normal samples and HNSCC samples without clinical information, the expression matrix was firstly assembled with the gene symbols as column and HNSCC samples as the row names. Following this, "WGCNA" package in R software was adopted to perform WGCNA^58^. The expression matrix was converted into an adjacency matrix that was further used to build an unsupervised co-expression relationship by using Pearson's correlation coefficients for all pair-wise genes. Based on scale-free topology criterion, we selected the best power (β = 4) as a soft-thresholding parameter to strengthen correlation adjacency matrix^59^. Secondly, the adjacency matrix was transformed into a topological matrix, and TOM was used to minimize interference from noise and spurious associations^60^. Any genes exhibiting identical expression profiles were grouped together into gene modules using hierarchical clustering. We set minimum module size as 30 genes. Finally, gene modules were identified from the system cluster tree by dynamic cutting algorithm and those with 70% similarity were merged into a module. Module eigengenes (MEs) were considered as the major component for each module, and together with the clinical information, they were used for MTR analysis. The MTR heat map was plotted to clearly visualize clinical trait related modules. Module significance (MS) was defined as the mean GS that was calculated based on the samples' clinical traits. Finally, the genes in the most significant module with strongest correlation to HNSCC clinical features were selected for further analysis.

### Construction of ceRNA networks

According to the ceRNA theory, we constructed lncRNA-miRNA-mRNA ceRNA networks with the following steps: (1) DEmiRNAs, co-expressed DElncRNAs and DEmRNAs in the most significant module were kept; (2) the interaction relationships between DElncRNAs and DEmiRNAs were predicted by miRcode (http://www.mircode.org); (3) the interaction relationships between DEmiRNAs and DEmRNAs were predicted by TargetScan (http://www.targetscan.org), miRTarBase (http://mirtarbase.mbc.nctu.edu.tw) and miRBase (http://www.mirbase.org); (4) overlapped DEmiRNAs which were negatively interacted with DElncRNAs and DEmRNAs were selected to constructed ceRNA networks. Then, the networks were visualized in Cytoscape software.

### Identification of HNSCC-specific prognostic lncRNA and mRNA signatures

In order to identify specific prognostic genes for HNSCC, we firstly performed KM curve analysis and LR test of DElncRNAs and DEmRNAs in ceRNA networks using "survival" package in R software. HNSCC patients were divided into high- and low-expression groups according to gene expression levels and LR *P* value < 0.05 was defined as the significant prognostic standard for lncRNAs and mRNAs. Then, univariate Cox proportional hazards regression analysis was implemented to evaluate the prognostic association of DElncRNAs and DEmRNAs in ceRNA networks in HNSCC patients by using "survival" package in R software as well. Statistical *P* value < 0.05 was set as the cut-off criteria. Next, multivariate Cox hazards regression model was constructed using overlapped DElncRNAs and DEmRNAs in the above two methods with "survival" package in R software, which were strongly significantly associated with OS of HNSCC patients. The multivariate Cox hazards regression model was built to evaluate the HNSCC-specific prognostic lncRNA and mRNA signatures. The model was established based on the following equation:

$$ {\text{Risk}}\;{\text{score}} = \sum \left( {Coe*Exp_{lncRNA/mRNA} } \right) $$

where "Coe" represented the regression coefficient of the lncRNA or mRNA retrieved from the multivariate Cox regression model and "Exp" referred to the expression level of the lncRNA or mRNA. Subsequently, HNSCC patients were divided into high- and low-risk groups based on the median risk score for all patients. A receiver operating characteristic (ROC) curve was plotted and AUC was calculated to access the risk prediction rate of risk model adopting "survivalROC" package in R software.

### Patients and tissue sample collection

Human OSCC samples were collected at The Affiliated Hospital of Stomatology, Zhejiang University School of Medicine between January 2018 and December 2020, which were applied to validate the expression difference of the genes. There were 6 males and 4 females (average age, 59.6 years old; range 45–76 years old). Table 4 shown the details. The tissue samples were immediately fixed in 4% paraformaldehyde, embedded in paraffin, sectioned, and processed for the following steps. Each experimental sample was separated into two parts, one was used for IHC analysis and one for total RNA extraction. Research was authorized by the Ethics Committee of The Affiliated Hospital of Stomatology, Zhejiang University School of Medicine. Informed consent was obtained from all participating patients. All experiments were carried out in accordance with the approved study and relevant guidelines.

### qPCR validation

Total RNA of tissue samples was extracted by TRIzol reagent (Invitrogen, USA) with following the manufacturer's instructions, quantified by applying NanoDrop 2000c spectrophotometer (Thermo Fisher Scientific, Inc., USA), and synthesized into cDNA by using RNeasy Mini Kit (Takara, Japan). qPCR analysis was performed through utilizing SYBR Green Master Mix (Takara, Kyoto, Japan) under the following conditions: 95 °C for 5 min; 45 cycles of 95 °C for 5 s and 60 °C for 30 s; one cycle of 95 °C for 5 s, 60 °C for 1 min and 95 °C for 15 s; and finally, 50 °C for 30 s. The primer sequences were listed in Supplementary Table 1. Relative expression levels of targeted mRNAs were measured using β‑actin as the internal control and analyzed using the 2^−∆∆Cq^ method.

### Immunohistochemistry analysis

The Human Protein Atlas (https://www.proteinatlas.org/) database was preliminarily employed to map tissue protein expression pattern for the identified genes^61^. Subsequently, paraffin embedded sections were deparaffinized in xylene and rehydrated in graded ethanol. After washing with distilled water, the sections were heated in 37 °C with pepsin for antigen recovery, then washed and incubated in 3% (v/v) hydrogen peroxide for 25 min to block endogenous peroxidase activity, and finally incubated in 3% (w/v) BSA. Sections were incubated with primary antibodies against MYL1 (1:100; SAB, 47440), ACTN2 (1:250; Proteintech, 14221-1-AP) and LAT (1:100; Proteintech, 11326-1-AP) overnight. Then, washing in PBS, the sections were incubated with horseradish peroxidase (HRP)-labeled secondary antibodies for 1 h at room temperature. Diaminobenzidine (DAB) was used for visualizing immunolabeling.

### Statistical analysis

The expression levels of lncRNAs and mRNAs in normal samples and HNSCC samples were firstly evaluated, and the association between expression levels and clinical stages were subsequently accessed. Besides, we also compared the genes' expression levels in high-risk group and low-group, and performed survival analysis by the LR test, of which the result was visualized by plotting KM curve. According to prognostic information of HNSCC patients, the comparison of expression in alive group and dead group was also analyzed. qPCR results were presented by using GraphPad Prism (version 7.0; GraphPad Software, Inc., USA). Student's t-test was used to compare two groups, and one-way ANOVA for more groups. All values are presented as the mean ± standard deviation and *P* < 0.05 was considered to indicate a statistically significant difference.

## Supplementary Information

Supplementary Information 1.

Supplementary Information 2.

## Data Availability

The HNSCC RNA-seq and miRNA-seq data were deposited in the TCGA database. Besides, please contact author for data and material requests.
