# Supplementary material for: A ceRNA-associated risk model predicts the poor prognosis for head and neck squamous cell carcinoma patients
Source: Sci Rep. 2021 Mar 18;11:6374. doi: 10.1038/s41598-021-86048-x (PMC7973582; doi:10.1038/s41598-021-86048-x)
Supplement: Supplementary file 1 — Supplementary Information 1. [file 41598_2021_86048_MOESM1_ESM.docx]

**Supplementary Table 1**. qPCR primers

| Gene | qPCR primer sequences |
| --- | --- |
| MYL1 | F: 5' - GTTGAGGGTCTGCGTGTCTTT  R: 5' - ACCCAGGGTGGCTAGAACA |
| ACTN2 | F: 5' - CAAACCTGACCGGGGAAAAAT  R: 5' - CTGAATAGCAAAGCGAAGGATGA |
| LAT | F: 5' - GATGAGGACGACTATCACAACCC  R: 5' - GAAGGCACTGTCTCGGATGC |
| β‑actin | F: 5' - ACTCTTCCAGCCTTCCTTCC  R: 5' - CGTCATACTCCTGCTTGCTG |
