# Supplementary material for: A ceRNA-associated risk model predicts the poor prognosis for head and neck squamous cell carcinoma patients
Source: Sci Rep. 2021 Mar 18;11:6374. doi: 10.1038/s41598-021-86048-x (PMC7973582; doi:10.1038/s41598-021-86048-x)
Supplement: Supplementary file 2 — Supplementary Information 2. [file 41598_2021_86048_MOESM2_ESM.docx]

**A ceRNA-associated risk model predicts the poor prognosis for head and neck squamous cell carcinoma patients**

**Short title: A ceRNA-associated risk model for HNSCC**

Yuzi Xu ^1,*^, Fengqin Xu ^3,*^, Yiming Lv ^4,*^, Siyuan Wang ^1^, Jia Li ^1^, Chuan Zhou ^1^, Jimin Jiang ^1^, Binbin Xie ^2,#^, Fuming He ^1,#^

^1^ Department of Oral Implantology and Prosthodontics, The Affiliated Hospital of Stomatology, School of Stomatology, Zhejiang University School of Medicine, and Key Laboratory of Oral Biomedical Research of Zhejiang Province, Hangzhou, Zhejiang, 310006, P.R. China

^2^ Department of Medical Oncology, Sir Run Run Shaw Hospital, Zhejiang University School of Medicine, Hangzhou, Zhejiang, 310016, P.R. China

^3^ The First Affiliated Hospital of Kangda College of Nanjing Medical University, The First People's Hospital of Lianyungang, The Affiliated Lianyungang Hospital of Xuzhou Medical University, Lianyungang, Jiangsu, 222000, P.R. China

^4^ Department of Colorectal Surgery, Sir Run Run Shaw Hospital, Zhejiang University School of Medicine, Hangzhou, Zhejiang, 310016, P.R. China

**#Correspondence to:** **Prof. Fuming He, DDS, MD.** Department of Oral Implantology and Prosthodontics, The Affiliated Hospital of Stomatology, School of Stomatology, Zhejiang University School of Medicine, and Key Laboratory of Oral Biomedical Research of Zhejiang Province, 395# Yanan Road, Hangzhou, Zhejiang, 310006, P.R. China. Email: hfm@zju.edu.cn. **Binbin Xie, PhD.** Department of Medical Oncology, Sir Run Run Shaw Hospital, Zhejiang University School of Medicine, 3# East Qingchun Road, Hangzhou, Zhejiang, 310016, P.R. China. Email: 387609530@qq.com.

***Equal contribution:** These authors contributed equally to this work.

*
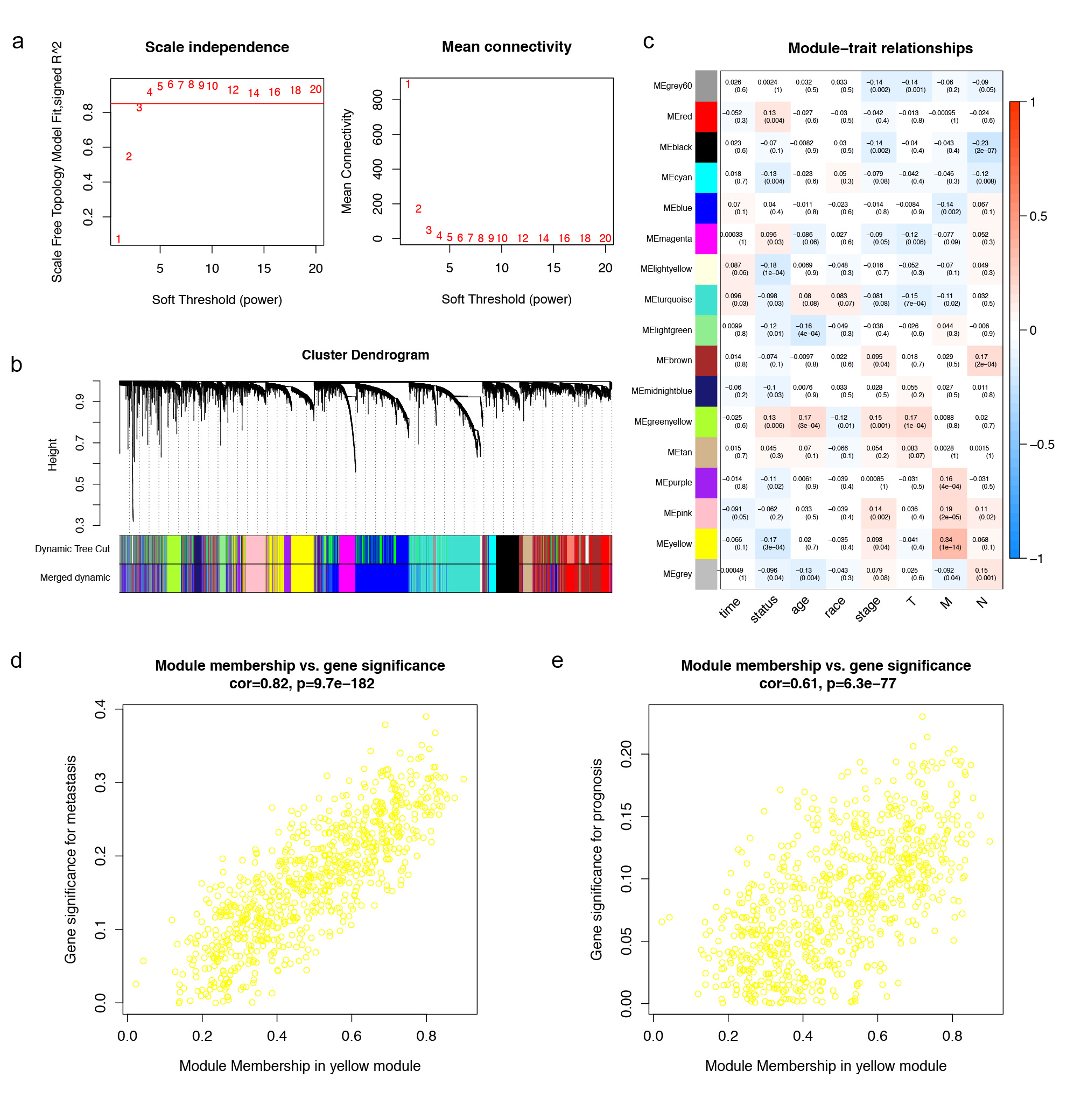
Supplementary figure 1.* Co-expression network analysis by WGNCA. (a) Soft-threshold power analysis for network topology. (b) Dendrogram of all DElncRNAs clustered based on a dissimilarity measure (1-TOM). (c) The relationships between modules and clinical traits. Yellow module was identified to be the most significant association with metastasis and prognosis in HNSCC patients. (d) The correlation between the yellow module membership and metastasis. (e) The correlation between the yellow module membership and prognosis.

*
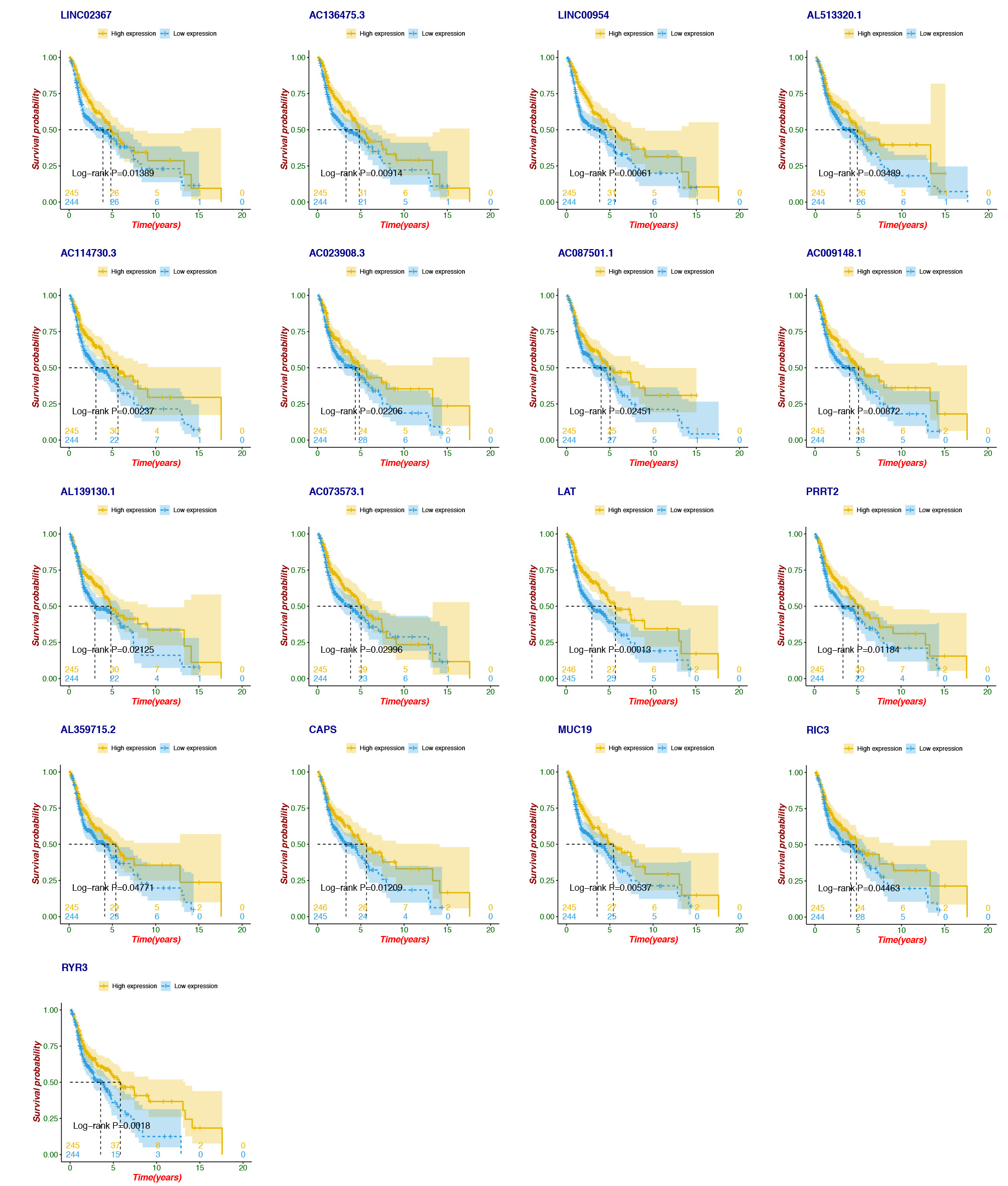
Supplementary figure 2.* Survival analysis. Low expression of 11 lncRNAs and 6 mRNAs predicted poorer OS than high expression in HNSCC patients.
